# Adaptive Personality Traits and Psychosocial Correlates among Living Kidney Donors

**DOI:** 10.3389/fpsyt.2017.00210

**Published:** 2017-10-23

**Authors:** Iris Pollmann, Faikah Gueler, Marie Mikuteit, Mariel Nöhre, Nicolas Richter, Karin Weissenborn, Martina de Zwaan

**Affiliations:** ^1^Department of Psychosomatic Medicine and Psychotherapy, Hannover Medical School, Hannover, Germany; ^2^Department of Nephrology, Hannover Medical School, Hannover, Germany; ^3^Department of Neurology, Hannover Medical School, Hannover, Germany; ^4^Department of Surgery, Hannover Medical School, Hannover, Germany

**Keywords:** living kidney donors, personality, NEO-Five Factor Inventory, fatigue, depression, anxiety

## Abstract

Since living kidney donors have repeatedly been shown to be mentally more healthy compared to the general population, they might also exhibit more adaptive personality characteristics. We investigated the personality traits of 315 living kidney donors (202 female and 113 male donors) on average 7.1 years after donation using the NEO-Five Factor Inventory, a frequently used personality inventory measuring the “big five” dimensions of personality (neuroticism, extraversion, openness, agreeableness, and conscientiousness). In addition, levels of depression, anxiety, and fatigue were assessed with the Patient Health Questionnaire-Depression Scale, GAD-7, and Multidimensional Fatigue Inventory. Kidney donors showed more adaptive personality traits with higher agreeableness and lower neuroticism scores compared to the German general population. This was even more pronounced in living kidney donors with a high motivation to donate again (non-regreters). Scores for depression, anxiety, and fatigue did not differ from general population values and were significantly correlated with most personality dimensions. The more adaptive personality characteristics of living kidney donors might either be a selection effect or the consequence of the experience of donation and improved health of the close relative. Regardless of the causal relationship, adaptive personality traits might positively influence both physical and psychosocial well-being of the donor. Longitudinal studies should investigate if living donation might lead to persistent adaptive changes in personality traits.

## Introduction

Worldwide, more than 20,000 kidney transplantations after living donation are performed each year (1). In Germany, 2,195 kidney transplantations were done in 2015, of which, 645 (29.4%) were from living kidney donors (2). In the same year, there were 7,961 transplantable patients on the waiting list showing the unfulfilled high demand for donor kidneys. The mean waiting period for kidney transplantation with deceased donor organs in Germany is estimated to be 6–7 years (2), but depends on the blood group of the recipients. For patients with blood group 0, the average waiting time is currently 9 years in Germany (3). Living kidney donation has become the gold standard treatment of end-stage renal failure. The benefits of kidney transplantation with living donor organs compared to deceased donor organs include pre-emptive transplantation, superior organ quality, longer graft survival, and improved event-free survival of the recipient (4).

Well-being following living kidney donation continues to be a concern and an important area of research. Even though recent studies have raised some concerns related to the long-term somatic safety of living donation (5, 6), experts agree that the long-term risks for the donor are generally low (7). Living donors are healthier with regard to somatic as well as psychological comorbidity than the general population, due to rigorous medical screening for kidney donor eligibility. This is supported by the finding that donors tend to have higher health-related quality of life (HRQoL) before donation compared to the general population (8). Studies indicate that shortly after donation during the early postoperative recovery period, donors have lower HRQoL with major changes in physical functioning and pain. HRQoL usually returns to baseline over time or remains slightly reduced compared to baseline, particularly for fatigue, but scores are still comparable to general population norms (8). Only a small group of donors report adverse psychosocial outcomes. The majority report no change or even an improved relationship with their recipient. Some donors experience an increase in self-esteem and a high degree of resilience, post-donate growth, and purpose in life (8–10). Almost all donors report that they would donate again supporting the living donation procedure (11). Thus, there is evidence that donors might even benefit psychologically from donation.

Little is known about the personality of individuals who chooses to consider living donation and if certain personality traits are common among living organ donors compared to the general population. One study investigated personality traits in 107 US living kidney donors, most of whom were 2–11 years after donation (10). They used the NEO-Five Factor Inventory (NEO-FFI) and found *T* scores (<45) in the low range for neuroticism and *T* scores (>55) for extraversion, agreeableness, and conscientiousness in the high range compared to age- and sex-specific population norms. The authors concluded that the donors in their sample showed a high degree of adaptive personality traits. However, the response rate was low, with only 16% of all kidney donors responding to the survey. It is perceivable that individuals with high agreeableness and conscientiousness might have been more willing to respond to a survey. Also, the cohort included 27% of kidney donors who were not part of the immediate family. Altruistic donors might have different personality profiles compared to donors with a close relationship to the recipients. German Transplantation Law does not permit altruistic donation and the personality profiles might, thus, be different in a German living kidney donors.

The aim of the study was to investigate personality traits in a large sample of German living kidney donors and to compare the results with a German norm population. In line with earlier studies, we expected to find more adaptive personality traits, specifically with regard to neuroticism, agreeableness, and conscientiousness. In addition, gender differences were investigated. Finally, correlations between personality traits and symptoms of depression, anxiety, and fatigue as well as the association with regret regarding donation were examined.

## Materials and Methods

### Participants

All individuals registered in the outpatient database of Hannover Medical School as living kidney donors where the donation was at least 1 year ago (1987–2016), and who were below 70 years of age at the time of the survey were contacted. They all received mailed packages of psychosocial assessment questionnaires and were asked to complete and return the packages in pre-addressed postage-paid envelopes that were provided. If patients did not respond, up to two new packages were sent out, each 6–8 weeks apart. In case of returned mail, new addresses were retrieved through administrative assistance of the registration offices and packages were sent to the donor’s last known address.

A cover letter was included explaining the aim of the study as well as a consent form. The study was approved by the Institutional Ethics Review Boards of Hannover Medical School and all patients gave written informed consent. Collected surveys were de-identified.

### Assessment Instruments

#### Personality

The NEO-FFI is a 60-item self-report measure of the “big five” dimensions of personality (12, 13). It consists of five 12-item scales (extraversion, neuroticism, agreeableness, conscientiousness, and openness to experience). Each item is rated on a 5-point scale from “strongly disagree” to “strongly agree.” “Extraversion” reflects characteristics such as social assertiveness, sociability, and sensitivity to positive emotions. Individuals with low “neuroticisms” are not easily distressed or sensitive to negative emotions; they are resilient in stressful situations and seldom experience feelings of anxiety, sadness, or depression. “Agreeableness” measures cooperativeness, altruism, and trust toward other people. “Conscientiousness” is expressed as self-control, orderliness, and adherence to social norms. “Openness to experience” correlates with curiosity, broad-ranging interests, and open-mindedness. The NEO-FFI has been used extensively and has demonstrated good internal consistency, test–retest reliability, and validity. Raw scores for each of the “big five” personality dimensions were transformed into sex- and age-specific standardized *T*-scores, which were based on the German population norm data published in the German manual (12). This is important since there are indications that personality can change to a certain extent with increasing age. *T*-scores for the NEO-FFI have a mean of 50; scores of 44 or less are in the “low” range according to NEO-FFI norms; scores of 56 or higher are in the “high” range; scores between 45 and 55 are considered “average” (13).

#### Symptoms of Depression and Anxiety

Symptoms of depression were assessed with the German version of the 9-item Patient Health Questionnaire-Depression Scale (PHQ-9) (14, 15). Each item is scored from 0 to 3, yielding a total score between 0 and 27. A total score ≥10 indicates the presence of a major depressive disorder. Cronbach’s α in the present study sample was 0.87. For the PHQ-9 data from a representative, population-based sample of 7,988 adults of 18–79 years old are available (16) and were used for comparison.

Symptoms of anxiety were assessed with the German version of the 7-item Generalized Anxiety Scale (GAD-7) (17, 18). The items are also scored from 0 to 3, yielding a total score between 0 and 21. A total score ≥10 indicates the presence of a clinically relevant anxiety disorder.

#### Fatigue

The Multidimensional Fatigue Inventory (MFI-20) (19, 20) contains 20 statements, which cover different aspects of fatigue. It comprises five subscales: general fatigue, physical fatigue, mental fatigue, reduced activity, and reduced motivation. Each subscale consists of four items to be answered on 5-point Likert scales ranging from “yes that is true” to “no that is not true.” Data from a representative sample of the German population aged 14–92 years (*n* = 2,037) are available for comparison (20). Data from the age groups 40–59 years were used for comparison with the kidney donors.

#### Sociodemographic and Donation-Specific Variables

The survey also contained investigator-generated questions on donor demographics, year of donation, the current relationship with the organ recipient, the donors’ willingness to donate again (regret), the current relationship with the organ recipient, and the donor’s own health.

### Statistics

IBM^®^ SPSS^®^ Statistics version 24.0.0.0 was used to carry out statistical analyses. Questionnaire scores were calculated for the entire kidney donor sample, and separate for male and female donors. Because questionnaire scores were not normally distributed (Shapiro–Wilk tests for all scores *p* < 0.05), median values and interquartile ranges (IQR) are reported in addition to means and SD. Mann–Whitney *U*-tests and chi-square tests were conducted to compare male and female donors and to compare donors with no regret regarding donation and donors with at least some regret. Two-sided Pearson correlations were conducted to investigate the association between the NEO-FFI subscales and levels of fatigue, depression, and anxiety.

## Results

**Figure 1:**
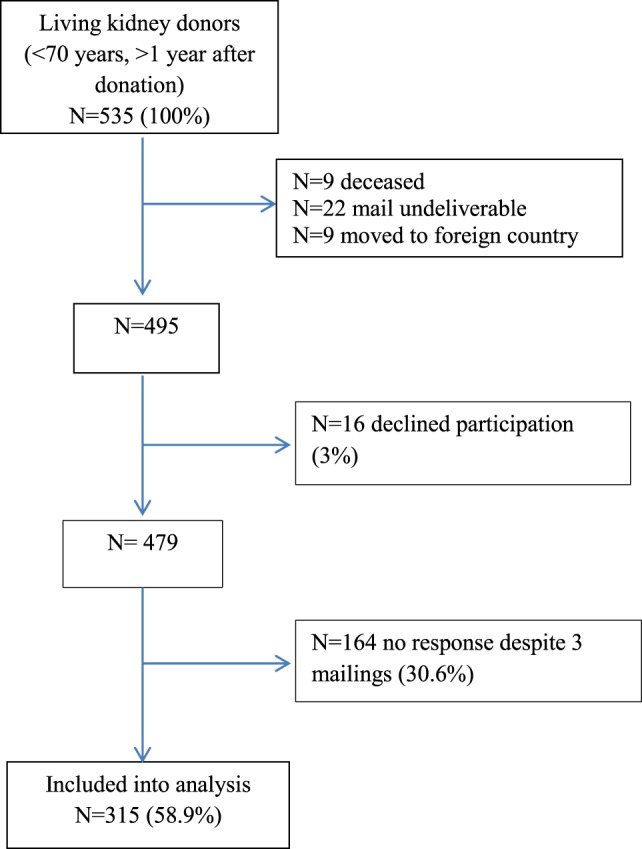
Study flow chart.

A total of 535 surveys were mailed to the selected living kidney donors. After numerous attempts, 315 were returned, for a response rate of 58.9% (Figure 1). Donors who did not return the survey did not differ significantly with regard to age [55.0 (SD 8.4) versus 55.9 (SD 8.0) years], sex (females 65.9 versus 64.1%), and relation to the organ recipient when compared with donors who returned the survey. However, years since donation differed significantly between participants and non-participants [8.24 (SD 5.2) versus 7.1 (SD 5.2) years; *T* = −2.297 (df = 477), *p* = 0.022].

**Figure 2:**
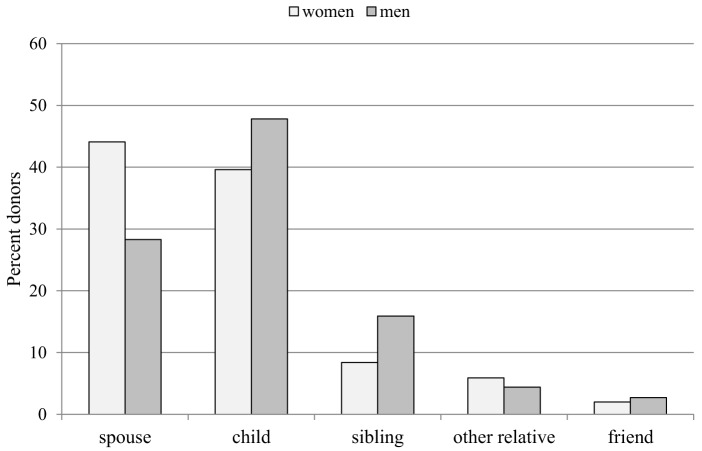
Relationship to the organ recipient by sex of the donor (percent donors).

Table 1 presents the demographic and clinical characteristics of the participants who completed the survey. Except for the relationship to the recipient, no characteristics differed between male and female donors. Male donors were more likely to have donated to a child (47.8%) whereas female donors more often donated to their spouse (44.1%) (Figure 2). Eighteen (5.7%) organ recipients had died. Of the 292 recipients who were alive (no information were available for 5 recipients), 20 (6.8%) had lost the donor’s kidney and 4 were on dialysis. Mean age at donation was 48.8 years (SD 8.8) ranging from 25 to 68 years [median 49 years (IQR 12)]. Years since donation ranged from 1 to 29 years with an average of 7.1 years (SD 5.2) [median 6 years (IQR 7)]. Overall, 239 (75.9%) donors reported no regrets at all whereas 73 (24.1%) reported at least some regret regarding donation. Of the latter 43 (13.7%) would likely donate again, 30 (9.6%) were unsure or would not choose to be a donor again, and 3 donors did not answer this question. 193 (61.3%) donors perceived their current relationship with the recipient as very good, 79 (25.1%) as good, 25 (7.9%) as neutral, bad, or very bad, and 18 did not answer this question. Finally, 69.9% donors described their own health as good to very good and 22.9% as moderate (Table 1).

**Table 1:** Sociodemographic and clinical characteristics of the study sample for the total sample and separate for male and female donors; comparison between sexes.

Characteristic	All survey respondents	Female donors	Male donors	*U*-test
	*N* = 315	*N* = 202	*N* = 113	χ^2^-test
Current age, mean (SD); range	55.9 (8.0); 29–69	55.8 (8.0); 33–69	56.1 (8.1); 29–69	ns

Educational level; ≥12 years of school attendance, % (*n*)	27.6 (87)	24.8 (50)	32.7 (37)	ns

Employment % (*n* = 305)				ns
Paid employment	72.1 (220)	71.2 (138)	73.9 (82)	
Retired/unemployed	27.8 (85)	28.2 (56)	26.1 (29)	

Partnership % (*n* = 314)	83.4 (262)	82.6 (166)	85.0 (96)	ns

Relationship to recipient, % (*n*)				χ^2^ = 9.973, df = 4, *p* = 0.041
Spouse	38.4 (121)	44.1 (89)	28.3 (32)	
Child	42.5 (134)	39.6 (80)	47.8 (54)	
Sibling	11.1 (35)	8.4 (17)	15.9 (18)	
Other relative	5.4 (17)	5.9 (12)	4.4 (5)	
Friends	2.2 (7)	2.0 (4)	2.7 (3)	
Undisclosed	0.3 (1)	0 (0)	0.9 (1)	

Age at donation, mean (SD); range	48.8 (8.8); 25–68	48.8 (8.8); 25–68	48.8 (8.7); 27–66	ns

Years since donation, mean (SD); range	7.1 (5.2); 1–29	7.0 (5.2); 1–26	7.3 (5.1); 1–29	ns

Years since donation % (*n*)				ns
<2	13.3 (42)	14.9 (30)	10.6 (12)	
2–6	40.6 (128)	39.1 (79)	43.4 (49)	
7–11	27.0 (85)	28.2 (57)	24.8 (28)	
>11	19.0 (60)	17.8 (36)	21.2 (24)	

Would you donate again?% (*n*)	75.9 (239)	77.7 (157)	72.6 (82)	ns
Definitely yes (non-regreters)				
Likely	13.7 (43)	12.9 (26)	15.0 (17)	
Unsure	4.8 (15)	4.5 (9)	5.3 (6)	
Not likely	3.2 (10)	2.5 (5)	4.4 (5)	
Definitely no	1.6 (5)	1.5 (3)	1.8 (2)	
Not answered	1.0 (3)	1.0 (2)	0.9 (1)	

How is your relationship to the recipient? % (*n*)				ns
Very good	61.3 (193)	60.9 (123)	61.9 (70)	
Good	25.1 (79)	24.8 (50)	25.7 (29)	
Moderate	6.3 (20)	6.4 (13)	6.2 (7)	
Bad	1.0 (3)	0.5 (1)	1.8 (2)	
Very bad	0.6 (2)	1.0 (2)	0.0 (0)	
Not answered	5.7 (18)	6.4 (13)	4.4 (5)	

Subjective health % (*n*)				ns
Very good	19.4 (61)	21.3 (43)	15.9 (18)	
Good	50.5 (159)	48.5 (98)	54.0 (61)	
Moderate	22.9 (72)	22.3 (45)	23.9 (27)	
Bad	5.7 (18)	5.9 (12)	5.3 (6)	
Very bad	0.6 (2)	0.5 (1)	0.9 (1)	
Not answered	10 (3)	1.5 (3)	–	

GAD-7 cutoff ≥10, % (*n*)	7.6 (24)	8.4 (17)	6.2 (7)	ns

Patient Health Questionnaire-Depression Scale cutoff ≥10, % (*n*)	10.2 (32)	11.4 (23)	8.0 (9)	ns

**Figure 3:**
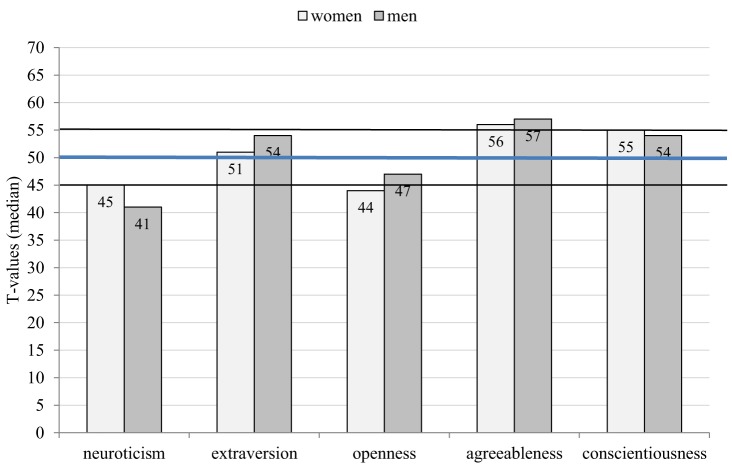
Median *T* scores on the NEO-Five Factor Inventory by sex of the donor (>55 is higher than average, <45 is lower than average).

*T*-scores for the five NEO-FFI dimensions were calculated for each participant. Compared to the German sex- and age-related population norms, the median values of the agreeableness *T* scores were above average (>55) in both male and female kidney donors. The median of the neuroticism *T* scores was below average (<45) in male donors and the median of the openness *T* scores was below average (<45) in female donors. All other *T* scores were in the average range (45–55) in both sexes. Overall, 49.4% (*n* = 154) reported neuroticism *T* scores below average and 51.3% (*n* = 160) reported agreeableness *T* scores above average. Male and female donors differed significantly on the neuroticism, extraversion, and openness *T* scores (Table 2; Figure 3).

**Table 2:** Means (SD), medians [interquartile ranges (IQR)], and sample sizes of psychosocial assessment instruments for the total sample and separate for male and female donors; comparison between sexes.

	All survey respondents			Female donors			Male donors			Mann–Whitney *U*-test
	Mean (SD)	Median (IQR)	Sample size	Mean (SD)	Median (IQR)	Sample size	Mean (SD)	Median (IQR)	Sample size	
Depression (PHQ-9)	3.9 (4.1)	3 (4)	315	4.4 (4.1)	3 (4)	202	3.0 (3.9)	2 (4)	113	*Z* = −3.925, *p* < 0.001
Anxiety (GAD-7)	3.4 (3.7)	2 (4)	315	3.7 (3.7)	3 (4)	202	2.7 (3.6)	2 (3.5)	113	*Z* = −3.240, *p* = 0.001
NEO-neuroticism	43.7 (11.6)	45 (17.75)	312	44.7 (11.3)	45 (16)	201	42.0 (12.0)	41 (19)	111	*Z* = −1.969, *p* = 0.049
NEO-extraversion	51.8 (11.0)	52 (13)	313	50.8 (10.9)	51 (13)	201	53.6 (11.1)	54 (13)	112	*Z* = −2.395, *p* = 0.017
NEO-openness	45.8 (9.4)	45 (13)	312	45.0 (9.1)	44 (13)	201	47.1 (9.8)	47 (13)	111	*Z* = −1.972, *p* = 0.049
NEO-agreeableness	54.5 (11.4)	56 (17)	312	54.0 (10.7)	56 (17)	201	55.3 (12.5)	57 (18)	111	*Z* = −1.101, *p* = 0.271
NEO-conscientiousness	55.0 (10.7)	55 (13.75)	312	54.9 (11.1)	55 (14.5)	201	55.4 (9.9)	54 (12)	111	*Z* = −0.348, *p* = 0.728
MFI-general	9.2 (4.2)	9 (6)	305	9.6 (4.3)	9 (6)	194	8.6 (3.9)	8 (6)	111	*Z* = −1.764, *p* = 0.078
MFI-physical	8.3 (3.9)	7.5 (6)	304	8.4 (4.0)	8 (6)	193	8.1 (3.9)	7 (6)	111	*Z* = −0.674, *p* = 0.500
MFI-mental	7.8 (4.0)	7 (6)	308	8.0 (4.2)	7 (6)	196	7.5 (3.8)	6.5 (5)	112	*Z* = −1.098, *p* = 0.272
MFI-activity	7.7 (3.8)	7 (5)	304	7.9 (4.1)	7 (6)	193	7.3 (3.2)	6 (4)	111	*Z* = −0.504, *p* = 0.614
MFI-motivation	7.2 (3.3)	6 (4)	307	7.3 (3.5)	6 (5)	195	7.1 (2.9)	6 (4)	112	*Z* = −0.120, *p* = 0.905

*PHQ-9, depression subscale of the Patient Health Questionnaire; GAD-7, Generalized Anxiety Subscale of the Patient Health Questionnaire; NEO, NEO-Five Factor Inventory; MFI, Multidimensional Fatigue Inventory-20*.

**Figure 4:**
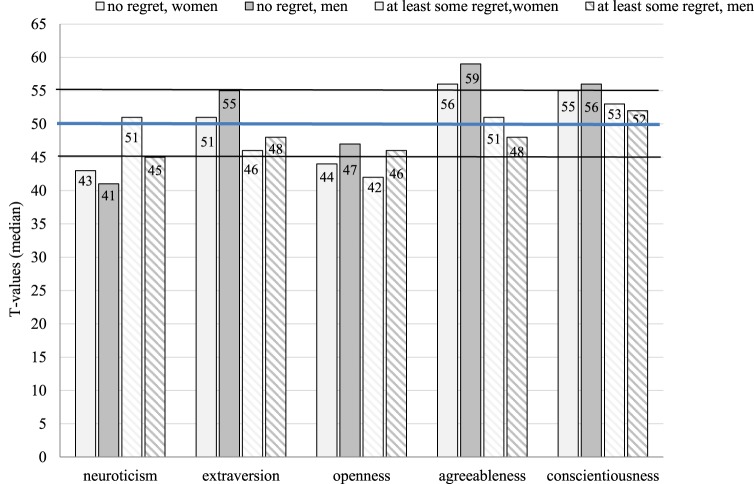
Median *T* scores on the NEO-Five Factor Inventory and regret regarding donation by sex (>55 is higher than average, <45 is lower than average).

Donors without any regret (*n* = 239) were compared with all other donors who reported at least some regret (*n* = 73). Donors without regret had significantly lower median neuroticism scores (*Z* = −3.973, *p* < 0.001) and significantly higher median values for extraversion (*Z* = −3.691, *p* < 0.001), agreeableness (*Z* = −3.555, *p* < 0.001), and conscientiousness (*Z* = −2.822, *p* = 0.005) compared to the remaining donors (Figure 4). These differences between groups were identified for both male and female donors with non-regreters showing more adaptive personality traits, which were frequently even below (neuroticism, openness) or above (agreeableness, conscientiousness) average levels in both sexes. 84.9% (*n* = 129) with neuroticism *T* scores below average reported no regret as opposed to 68.8% (*n* = 108) with *T* scores within the normal range or above average [χ^2^ = 11.172 (df = 1), *p* = 0.001]. The respective percentages for agreeableness *T* scores were 83.6% (>55) versus 69.3% (≤55) [χ^2^ = 8.849 (df = 1), *p* = 0.003].

We found no differences in personality profiles between genetically and emotionally linked donors (data not shown).

**Figure 5:**
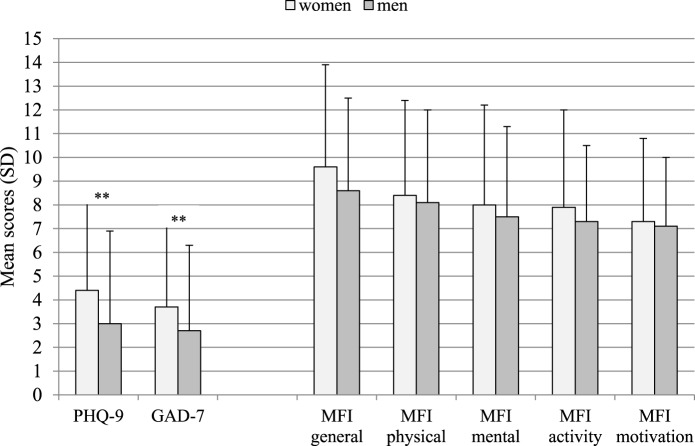
Mean scores and SDs on the Patient Health Questionnaire-Depression Scale (PHQ-9), GAD-7, and Multidimensional Fatigue Inventory subscales by sex. **Mann–Whitney *U*-test: *p* ≤ 0.001. PHQ-9, Depression subscale of the Patient Health Questionnaire; GAD-7, Generalized Anxiety Subscale of the Patient Health Questionnaire; MFI, Multidimensional Fatigue Inventory-20.

Symptoms of depression and anxiety were within the normal range and the average values were far away from the cutoffs of ≥10; however, 7.6% of the sample exhibited values above the cutoff on the anxiety scale and 10.2% on the depression scale (Table 3; Figure 5). Female donors had significantly higher depression and anxiety scores compared to male donors (Table 2); however, the percentage of patients above the cutoff score of 10 did not differ between sexes and were comparable to population values (16).

**Table 3:** Prevalence of depressive symptoms (Patient Health Questionnaire-Depression Scale ≥10) in percent with 95% confidence intervals (CI) for living kidney donors and comparison with a general population sample of 7,524 individuals Busch et al. (16).

	General population % (95% CI)	Kidney donors % (95% CI)
Women	10.2 (8.9–11.5)	11.4 (7.4–15.6)
Men	6.1 (5.2–7.2)	8.0 (3.4–13.0)
Total	8.1 (7.3–9.1)	10.2 (6.6–13.6)

Scores on the 5 subscales of the MFI-20 did not differ between sexes (Table 2; Figure 5) and were similar to population norms. When comparing the mean values between the donors and a sample of 693 individuals aged 40–59 years from the general German population (20), effect sizes (Cohen’s *d*) for mean values were low, not exceeding 0.2. This was true for both sexes.

Except for openness, the NEO-FFI dimensions were significantly correlated with the levels of depression, anxiety, and fatigue. While neuroticism was negatively correlated, extraversion, agreeableness, and conscientiousness were positively correlated (Table 4). However, only for neuroticism the correlation coefficients were >0.5 (but <0.64) indicating a relationship of moderate strength. All other correlation coefficients were <0.5 indicating a weaker association.

**Table 4:** Pearson correlations between NEO-Five Factor Inventory scales (median *T* scores) and levels of fatigue, anxiety, and depression.

		MFI general	MFI physical	MFI activity	MFI motivation	MFI mental	GAD anxiety	PHQ depression
NEO neuroticism	*r*	0.540	0.523	0.520	0.547	0.535	0.639	0.614
	*p*	0.000	0.000	0.000	0.000	0.000	0.000	0.000
	*N*	302	303	301	304	305	312	312

NEO extraversion	*r*	−0.474	−0.487	−0.457	−0.510	−0.433	−0.459	−0.509
	*p*	0.000	0.000	0.000	0.000	0.000	0.000	0.000
	*N*	303	303	302	305	306	313	313

NEO openness	*r*	−0.058	−0.008	−0.011	−0.103	−0.089	−0.047	−0.070
	*p*	0.312	0.896	0.852	0.073	0.121	0.413	0.215
	*N*	302	303	301	304	305	312	312

NEO agreeableness	*r*	−0.217	−0.200	−0.166	−0.233	−0.156	−0.322	−0.266
	*p*	0.000	0.000	0.004	0.000	0.006	0.000	0.000
	*N*	302	303	301	304	305	312	312

NEO conscientiousness	*r*	−0.295	−0.347	−0.464	−0.416	−0.393	−0.330	−0.372
	*p*	0.000	0.000	0.000	0.000	0.000	0.000	0.000
	*N*	302	303	301	304	305	312	312

*Pearson correlations two-sided*.

*PHQ-9, Depression subscale of the Patient Health Questionnaire; GAD-7, Generalized Anxiety Subscale of the Patient Health Questionnaire; NEO, NEO-Five Factor Inventory; MFI, Multidimensional Fatigue Inventory-20*.

Age of the respondents, age at time of donation, and duration since donation did not reveal significant associations with personality traits, levels of depression, anxiety, or fatigue (data not shown).

## Discussion

We identified specific personality characteristics in a large group of living kidney donors with higher than average levels of agreeableness in both sexes and lower than average levels of neuroticism in men and of openness in women. It is difficult to interpret the gender differences; however, the trend was in the same direction. These results were more pronounced in donors without any regret regarding donation. High agreeableness is characterized by good-naturedness, cooperativeness, and an overall prosocial orientation (forgiving, not demanding). Low neuroticism, on the other hand, is characterized by emotional stability and low vulnerability to stress. Overall, the results regarding agreeableness and neuroticism are similar, even though less pronounced, to the results of an earlier study in 107 kidney donors (10). Even though altruistic donation is not permitted in Germany and cross donation is hardly done, the personality profile resembles the profile found within the U.S. living kidney donors where these options are allowed. As opposed to this study, we did not find higher than average conscientiousness levels. As mentioned before, participation rate in the US study was only 16%, which may have resulted in a selection bias.

These results can be interpreted in different ways. More adaptive personality characteristics may influence the process of decision making and individuals with low neuroticism and high agreeableness might be more willing to donate. Specifically, agreeableness and neuroticism play an important role in social contexts and influence social interactions. Also, it is perceivable that individuals who describe themselves as altruistic and emotionally stable are more likely to successfully complete the screening process and to realize the living kidney donation. However, it remains unclear if individuals with a more adaptive personality profile are more willing to donate an organ or if there might be a selection bias of potential donors in the prescreening process with the acceptance of more “healthy” individuals as living donors. This is supported by a study of Erim et al. (11) who reported that living kidney donors who were excluded from donation after the screening process due to donor health related issues had significantly lower levels of resilience compared to eligible donors.

There is also an alternative explanation. Although personality is conceptualized as a stable dispositional behavioral trait, environmental factors have shown to influence stability and change of personality traits. This has been most extensively investigated for neuroticism (21). There is clear evidence from longitudinal studies that positive live events such as—among others—recovery from serious illness of a family member are associated with small but lasting decreases in neuroticism. Thus, the low levels of neuroticism in our male sample might be explained by a true and lasting shift in neuroticism caused by the improvement of the health of a close family member. Others have reported that over an 8-year period, changes of all five NEO-FFI personality dimensions were significantly associated with changes in different measures of perceived social support (22). There is also evidence that relationship satisfaction is not only associated with decreases of neuroticism but also with small and lasting increases in extraversion over time (23). In addition, studies have shown (24) that there seems to be a reciprocal effect in that decreases in neuroticism may evoke positive experiences, which might lead to a benign cycle. Low neuroticism and positive experiences may reinforce each other in daily life. This might not only be of benefit for the donor but also for the relationship between donor and recipient. However, to what extent and in whom donation might lead to persistent personality changes is currently unsettled and can only be answered in prospective long-term studies.

Overall, levels of fatigue were comparable to the general German population and to an earlier German study including 295 living kidney donors (20, 25). Also, levels of depression and anxiety were comparable to German norms (16). Compared to a similar study in a German cohort of 295 live kidney donors (25), the mean depression scores on the PHQ-9 were comparable with 3.9 (SD 4.1) in our study and 3.59 (SD 3.99) in Sommerer et al (25). In our study, 10.2% of the donors met the cutoff (PHQ-9 ≥ 10) for depressive disorder, which was somewhat higher compared to the 2.1% reported by Sommerer et al. (25) but was within the range of 5–23% summarized in a systematic review (9). Female donors reported higher depression and anxiety scores compared to male donors; however, the percentage of donors with values above the cutoff (≥10) did not differ between sexes. The finding of a lack of difference with population values is in line with the majority of studies investigating quality of life, fatigue, and mental health post-donation (8, 9, 25–27). However, as with somatic complications, comparisons with general population samples are biased, since there is strong evidence that living kidney donors are physically and mentally healthier than the general population (8). Kroencke et al. (26) reported better HRQoL in living kidney donors compared with the general population, but comparable scores compared with healthy controls prior to donation and 1-year post-donation. General population samples might skew results in favor of the donor cohort for both somatic complications (27) and psychological sequelae (26). Post-donation comparisons with general population samples might even hide a negative outcome.

More adaptive personality characteristics were significantly associated with lower levels of fatigue, depression, and anxiety. This was true for all NEO-FFI dimensions except for openness to new experiences. In a meta-analysis of 10 prospective cohort studies low extraversion, high neuroticism, and low conscientiousness were found to be associated with depressive symptoms both cross-sectionally and also longitudinally (28). Personality traits, specifically, neuroticism seem to be robust predictors of vulnerability not only for mental health disorders but also for physical illnesses (29) and even predict longevity (after controlling for putative confounders). It has been argued that the economic burden of neuroticism exceeds that of common mental disorders and those of somatic disorders (30, 31). Hence, an adaptive personality style, either already present prior to donation or developing after donation, might protect against further mental complications and contribute to stable psychosocial health following donation.

This study contains a large group (*n* = 315) of living kidney donors with an acceptable response rate of 59% compared to the only other study that used the NEO-FFI in a donor sample (16% in 10), which improves the generalizability of our results. In addition, the NEO-FFI allows the calculation of sex- and age-specific standardized *T*-scores, which facilitates comparisons with population samples.

Several considerations may limit the interpretation of our findings. It is a cross-sectional study, there are no baseline questionnaire data prior to donation available and we do not know if there were within-subject changes after donation. More than 30% of the donors have not responded to the survey and a social desirability bias cannot be excluded. More agreeable donors might have been more willing to participate in the survey. However, except for time since donation, there were no differences between participants and non-participants at least with regard to age, sex, and organ recipients.

Future studies should consider ethnicity and immigrant status when investigating personality dimensions in organ donors. Finally, there is evidence that empathic concerns might predict donation willingness (32). Even though the personality dimension agreeableness shows a high correlation with empathy (33), the constructs are not entirely overlapping. The role of empathy in organ donors has not been investigated in depth as yet.

### Conclusion

The current study provides evidence to suggest that living kidney donors exhibit more adaptive personality traits, specifically, high agreeableness and low neuroticism compared to age- and sex-specific population norms. Adaptive personality traits might be a protective factor for the donors’ own physical and mental health and might improve the relationship with the recipient. However, we cannot differentiate if the adaptive personality characteristics were already present prior to donation or if they developed as a result of the donation experience. To establish causal associations a longitudinal design is necessary. For future cross-sectional studies, a comparison with healthy population samples might be preferable since donors are inherently healthier at baseline than the general population.

## Ethics Statement

The study was approved by the Ethics committee of Hannover Medical School (no 3252-2016) and the participants signed an informed consent.

## Author Contributions

MZ, KW, and FG designed the study and received funding. All authors contributed substantially to the acquisition and interpretation of the data. MZ and IP analyzed the data and MZ and MN wrote the first draft. All authors critically revised the manuscript and gave their final approval. All authors gave their final approval of the version to be published.

## Conflict of Interest Statement

All authors declare that the research was conducted in the absence of any commercial or financial relationship that could be construed as a potential conflict of interest.
